# Use of Ethanol in the Trans-Arterial Lipiodol Embolization (TAELE) of Intermediated-Stage HCC: Is This Safer than Conventional Trans-Arterial Chemo-Embolization (c-TACE)?

**DOI:** 10.1371/journal.pone.0129573

**Published:** 2015-06-25

**Authors:** Francesco Somma, Roberto D’Angelo, Nicola Serra, Gianluca Gatta, Roberto Grassi, Francesco Fiore

**Affiliations:** 1 Department of Medicine and Surgery “Magrassi and Lanzara”, Section of Radiology, Second University of Naples (SUN), Napoli, Italy; 2 Department of Interventional Radiology, National Cancer Institute of Naples “Fondazione Pascale”, Napoli, Italy; University Hospital of Essen, GERMANY

## Abstract

**Purpose:**

To evaluate safety and efficacy of Trans-Arterial Ethanol-Lipiodol Embolization (TAELE) compared with conventional Trans-Arterial Chemo-Embolization (cTACE) in the treatment of small intermediate-HCC (BCLC-Stage B).

**Materials and Methods:**

A random sample of 87 patients (37.93% male; 62.07% female; age range, 36–86 years) with documented small intermediate-HCC and treated with TAELE (mixture 1:1 of Ethanol and Lipiodol) or cTACE (mixture of 50mg-Epirubicin and 5cc-Lipiodol) were retrospectively studied in an institutional review board approved protocol. The two procedures were compared with χ^2^-test, χ^2^-test with Yates correction, McNemar’s exact test, ANOVA test and log-rank test.

**Results:**

TAELE and cTACE therapies were performed in 45 and 42 patients, respectively. Thirty days after the procedure, a Multi-Detector Computed Tomography (MDCT) showed no significant difference in the number of patients with partial and complete response between the two groups (p-value = 0.958), according to mRECIST. Contrary, significant differences were found in tumor-devascularization, lesion-reduction and post-embolization syndrome occurrence (p-value = 0.0004, p-value = 0.0003 and p-value = 0.009, respectively). Similar survival was observed during 36-month follow-up (p-value = 0.884).

**Conclusion:**

Compared to cTACE, TAELE showed a better toxicity profile with similar 36-month survival and similar one-month anti-tumor effects, which makes it better tolerated by patients, especially in case of more than one treatment.

## Introduction

Hepatocellular carcinoma (HCC) is a major health problem worldwide, affecting more than 600,000 new patients per year [1]. Chronic liver disease of various etiology predisposes to HCC, which ranks as the first cause of death among cirrhotic patients [2]. Curative treatments are hepatic resection, liver transplantation and percutaneous ablation (RFA) [3]. Although surveillance programs have led in Western countries to an increase in the applicability of radical therapies that nowadays are indicated in 30%-40% of patients [4], the majority of them are not treatable with surgical resection due to their advanced stage at presentation [5]. In these cases, it is compulsory to find alternative treatment or palliative therapies.

Many patients not eligible to curative surgery have a locally advanced, liver restricted disease, known as intermediate HCC according to Barcelona Clinic Liver Cancer (BCLC). Since no extra-hepatic spread is present at this stage, trans-arterial loco-regional modalities offer palliative treatment options in these patients. Such therapies exploit the dual blood supply to liver, since HCC derives its blood supply almost entirely from hepatic artery, while the liver parenchyma derives more than 75% of its blood supply from the portal vein [6]. This anatomical fact is the basis for the development of intra-arterial treatments of HCC, inducing a selective tumor necrosis while sparing the surrounding parenchyma. So far, according to the BCLC staging system, conventional trans-arterial chemo-embolization (cTACE) has been recommended as the standard of care only for intermediate stage HCC, while the advanced stage is still seen as a contraindication, since cTACE has been proved to increase the risk of complications, including acute liver failure or intra-hepatic tumor progression [7]. Indeed, many protocols of trans-arterial treatment result in a characteristic syndrome following embolization, known as post-embolization syndrome, that occurs in 60–80% of patients and consists of fatigue, transient abdominal pain, ileus, fever and increased serum levels of liver enzymes and bilirubin [8]. On the other hand, transcatheter embolizing procedures using Lipiodol and Ethanol have already been described for HCC [9–13], assessing that the efficacy and treatment effectiveness were probably even superior to those of chemoembolization. However, to our knowledge, none of these studies has focused on the procedure-related toxicity, with particular regard to post-embolization syndrome.

Therefore, the aim of this study is to determine the efficacy and the safety of the Trans-Arterial Ethanol-Lipiodol Embolization (TAELE), using a mixture 1:1 of Ethanol and Lipiodol, compared with cTACE in the treatment of intermediate HCC (Stage B according to BCLC). This protocol avoids the administration of chemotherapeutic agents, thus reducing side effects due to the use of anti-tumoral drugs.

## Material and Methods

### Ethics Statements

This study has been approved by the Scientific Committee of the National Cancer Institute "Fondazione Pascale", Via Mariano Semmola, 80131 Napoli. Appropriate written informed consent was collected before each procedure.

### Patients

Between January 2009 and December 2010, all patients undergoing trans-arterial procedures for HCC at our Interventional Radiology Department were considered. Inclusion criteria were intermediate stage HCC (BCLC Stage B) with liver restricted disease, histologically confirmed by percutaneous image-guided liver biopsy and the treatment with a trans-arterial procedure (both TAELE or cTACE). Patients with multiple trans-arterial treatments were only considered at their first procedure. Extra-hepatic spreading disease and prior trans-arterial procedures (including radioembolization) were considered as exclusion criteria. 87 patients (33 M, 54 F; age range, 36–86y) with intermediate stage HCC and treated with cTACE or TAELE were eventually included. Data were retrospectively collected by a dedicated data manager in an institutional review board approved protocol. 45 patients underwent TAELE, while the remaining 42 underwent cTACE. The decision of which treatment to perform had been taken case by case by a multidisciplinary team on the basis of various parameters, above all the resistance to previous locoregional treatments and impaired liver function (patients with moderate ascites, increased serum bilirubin, reduced serum albumin). Further technical considerations were even taken into account. One month after the interventional procedure, an abdominal contrast enhanced Multi-detector Computed Tomography (MDCT) assessment scan control was performed in all patients. At least 36-month follow-up was carried out for patients in both groups. Only one patients treated with cTACE was lost at follow-up after 35 months. Abdominal images were reviewed by a 17-year liver experienced radiologist, blinded to the trans-arterial therapy performed. Written informed consent was obtained from all patients before each procedure.

### TAELE

45 patients (17 M, 28 F; age range, 36–86y; mean age about 62y with standard deviation about 10y) underwent TAELE: using a standard angiographic approach, transfemoral visceral arteriography was performed. All embolizing procedures were realized by advancing a micro-catheter (Progreat and Progreat Omega, Terumo Corporation, Tokyo, Japan) with a 2.4-F tip or a 2.7-F tip through a 4-F and 5-F catheter (Imager II C1-Selective and RC2-Selective, Boston Scientific Corporation, Natick, MA, USA), into the branches of the hepatic arteries feeding the tumor. A super-selective catheterization of the tumor-feeding branches was attempted in all cases; however, the catheter and micro-catheter were positioned differently according to the tumor distributions and anatomical variations. To avoid the vasospasm, intra-arterial slow administered of 2ml of Lidocaine (Galenica Senesa, Siena, Italy) diluted with 10 mL saline were routinely administered. Afterwards, a mixture of 1:1 Lipiodol (Guebert SpA, Genova, Italy) and Ethanol (99.5%) was injected continuously at the rate of 0.5 to 1.0 mL/min, followed by the administration of 45–150μ polyvinyl-alcohol (PVA) embolizing particles (Contour 45–150μ, Boston Scientific Corporation, Natick, MA, USA) until the site was filled.

### c-TACE

42 patients (16 M, 26 F; age range, 48–77y; mean age about 64y with standard deviation about 6y) underwent cTACE: under fluoroscopic guidance, a selective catheterization of the tumor-feeding arteries was obtained, similarly to that previously described for TAELE. After the intra-arterial injection of Lidocaine (Galenica Senesa, Siena, Italy), a combination of 5mL of Lipiodol (Guebert SpA, Genova, Italy) and 50mg of Epirubicine (Epirubicin Hydrochloride Powder for Injection, Pfizer Ltd, Sandwich, United Kingdom) was delivered through the micro-catheter directly into the tumor, followed by the administration of PVA embolizing particles, until the site was filled.

### Response Evaluation and Toxicity

Radiological response was evaluated according to the well established modified Response Evaluation Criteria in Solid Tumors (mRECIST) and the European Association for the Study of the Liver (EASL) guidelines [14,15], using a contrast enhanced MDCT in dual phase acquisition (arterial phase and venous phase) [16]. In particular, the overall tumor response to TAELE or cTACE was evaluated case by case according to mRECIST as follows: Complete Response (CR), Partial Response (PR), Progressive Disease (PD) and Stable Disease (SD). Moreover, the degree of vascularization of tumor lesions was evaluated by comparing contrast-enhanced MDCT images at baseline with those performed one month after the procedure [17]. Generally, by comparing the density of the liver parenchyma in the enhanced phase of acquisition, the following features could be observed after TAELE or cTACE: high vascularization with presence of persistent flow, hyperdensity of the treated lesion and no uptake of lipiodol; partial devascularization with areas of persistent vascularization alternated with areas of absent vascularization and partial uptake of lipiodol; absent vascularization with hypodense lesion and high uptake of lipiodol. Furthermore, laboratory data including serum total bilirubin (normal range 0.2–1.1 mg/mL), aspartate aminotransferase (AST, normal range: 10–40 U/L), alanine aminotransferase (ALT, normal range 10–40 U/L) were examined 1 day before the procedure, 2-, 4-, 6- and 30 days after the procedure in order to monitor the liver toxicity of each procedure. The safety of both procedures was assessed considering the frequency of adverse events up to 4 weeks following the treatment according to the National Cancer Institute’s Common Terminology Criteria for adverse events (CTCAE version 4.0, 2009). Thus, we considered as adverse events the following side effects: Grade 1—abdominal pain, nausea, vomiting, fatigue and fever; Grade 2—thrombocytopenia, leukocytosis, transient increase in liver enzymes, and LDH; Grade 3—acute renal failure, hypoxia, remarkable increase in serum total bilirubin and liver enzymes, severe arterial hypertension; Grade 4—life-threatening consequences of the procedure with urgent operative intervention indicated; Grade 5—death.

### Statistical analysis

The statistical analyses were performed using Matlab statistical toolbox version 2008 (MathWorks, Natick, MA, USA) for Windows at 32 bit, on two groups of patients with similar characteristics in terms of sex and age (three intervals were considered: [36–52], [53–69] and [70–86], with percentages of 13.33%, 62.22% and 24.44% in TAELE group; 2.38%, 78.57% and 19.05% in cTACE group). All tests to compare TAELE and cTATE were performed using the χ^2^ test [18] or χ^2^ test with Yates correction [18], the analysis of variance test (ANOVA) [19] and the McNemar’s exact test [20]. Eventually, survival curves were assessed with Kaplan-Meier method [21,22] and compared using the log-rank test [21]. All tests with p-value < 0.05 were considered as significant.

## Results

The following time-points were fixed: T_0_—baseline time before the procedure; T_1_—one week after the procedure; T_2_—one month after the procedure. The baseline population characteristics were listed in Table 1. At baseline, no significant differences were noticed between TAELE and cTACE groups in terms of sex and age (respectively p-value = 0.849 and p-value = 0.113, χ^2^ test) and tumor burden calculed on the baseline sum diameters (p-value = 0.0748, ANOVA test), as shown in Table 2.

**Table 1:** Baseline (T_0_) Demographics in Patients with HCC with CTCAE version 4.0 Toxicities.

Parameters	TAELE (n = 45)	cTACE (n = 42)
***Age*** (y)		
<65	24/45 (53.33)	22/42 (52.38)
≥65	21/45 (46.67)	20/42 (47.62)
***Sex***		
Male	17/45 (37.78)	16/42 (38.1)
Female	28/45 (62.22)	26/42 (61.9)
***Cause***		
Hepatitis C virus	23/45 (51.11)	25/42 (59.52)
Hepatitis B virus	8/45 (17,78)	6/42 (14.29)
Alcohol	4/45 (8.89)	6/42 (14.29)
Hepatitis C and B viruses	3/45 (6.67)	2/42 (4.76)
Autoimmune	2/45 (4.44)	0/42 (0)
Cryptogenetic	1/45 (2.22)	0/42 (0)
NASH	1/45 (2.22)	1/42 (2.38)
Unknown	3/45 (6.67)	2/42 (4.76)
***ECOG performance status***		
0	27/45 (60)	29/42 (69.05)
1	16/45 (35.56)	12/42 (28.57)
2	2/45 (4.44)	1/42 (2.38)
***Prior Treatment***		
None	33/45 (73.33)	35/42 (83.33)
Resection	3/45 (6.67)	2/42 (4.76)
RFA	6/45 (13.33)	4/42 (9.52)
PEI	3/45 (6.67)	1/42 (2.38)

**Note.** Data in parentheses are percentages. NASH = nonalcoholic steatohepatitis. ECOG = Eastern Cooperative Oncology Group. RFA = radiofrequency ablation. PEI = percutaneous ethanol injection. AST = aspartate aminotransferase. ALT = alanine aminotransferase.

**Table 2:** Statistical tests: TAELE vs. cTACE.

Test characteristics	Hypothesis	Test type:	P-value
Sex	28(F)/17(M) > 26(F)/16(M)	_χ_^2^ with Yates correction	0.849
Age	TAELE group ≠ cTACE group	_χ_^2^	0.113
Therapy response (CR-PR-SD-PD)	TAELE ≠ cTACE	_χ_^2^ with Yates correction	0.958
Nr.of patients with adverse events	42.22% (TAELE) < 57.14% (cTACE)	_χ_^2^ with Yates correction	0.239
Nr. of adverse events	7.78% (TAELE) < 14.29% (cTACE)	_χ_^2^ with Yates correction	0.009
Tumoral mean size at T_0_ (TAELE vs cTACE)	*μ*_1_ = 2.864cm > *μ*_2_ = 2.486cm	ANOVA	0.0748
Tumoral reduction in size in T_0_ –T_2_ (TAELE vs cTACE)	*μ*_3_ = 1.384cm > *μ*_4_ = 1.064cm	ANOVA	0.0003
Tumor devascularization in T_0_-T_2_ (TAELE vs cTACE)	*μ*_5_ = 2.511cm > *μ*_6_ = 1.955cm	ANOVA	0.0004
Survival time at 36 months	TAELE ≠ cTACE	log-rank test	0.884
Nr. patients in TAELE group with abnormal values of AST.	71.11% (T_0_) < 80.00% (T_1_)	McNemar’s exact test	0.109
Nr. patients in TAELE group with abnormal values of AST.	80.00% (T_1_) > 60.00% (T_2_)	McNemar’s exact test	0.0176
Nr. patients in TAELE group with abnormal values of AST.	71.11% (T_0_) > 60.00% (T_2_)	McNemar’s exact test	0.113
Nr. patients in c-TACE group with abnormal values of AST.	64.29% (T_0_) < 85.71% (T_1_)	McNemar’s exact test	0.0112
Nr. patients in c-TACE group with abnormal values of AST.	85.71% (T_1_) > 80.95% (T_2_)	McNemar’s exact test	0.363
Nr. patients in c-TACE group with abnormal values of AST.	64.29% (T_0_) < 80.95% (T_2_)	McNemar’s exact test	0.0195
Nr. patients in TAELE group with abnormal values of ALT.	60.00% (T_0_) < 68.89% (T_1_)	McNemar’s exact test	0.1093
Nr. patients in TAELE group with abnormal values of ALT.	68.89% (T_1_) > 48.49% (T_2_)	McNemar’s exact test	0.0059
Nr. patients in TAELE group with abnormal values of ALT.	60.00% (T_0_) > 48.89% (T_2_)	McNemar’s exact test	0.0625
Nr. patients in c-TACE group with abnormal values of ALT.	54.76% (T_0_) < 78.57% (T_1_)	McNemar’s exact test	0.001
Nr. patients in c-TACE group with abnormal values of ALT.	78.57% (T_1_) > 73.81% (T_2_)	McNemar’s exact test	0.312
Nr. patients in c-TACE group with abnormal values of ALT.	54.76% (T_0_) < 73.81% (T_2_)	McNemar’s exact test	0.0107
Nr. patients in TAELE group with abnormal values of bilirubin.	60.00% (T_0_) = 60.00% (T_1_)	McNemar’s exact test	0.637
Nr. patients in TAELE group with abnormal values of bilirubin.	60.00% (T_1_) > 44.44% (T_2_)	McNemar’s exact test	0.0195
Nr. patients in TAELE group with abnormal values of bilirubin.	60.00% (T_0_) > 44.44% (T_2_)	McNemar’s exact test	0.0461
Nr. patients in c-TACE group with abnormal values of bilirubin.	33.33% (T_0_) < 64.29% (T_1_)	McNemar’s exact test	0.0001
Nr. patients in c-TACE group with abnormal values of bilirubin.	64.29% (T_1_) > 57.14% (T_2_)	McNemar’s exact test	0.187
Nr. patients in c-TACE group with abnormal values of bilirubin.	33.33% (T_0_) < 57.14% (T_2_)	McNemar’s exact test	0.0032

**Note.** T_0_ = baseline. T_1_ = within the first week after the procedure. T_2_ = one month after the procedure.

*μ*_1_ = mean tumoral size at T_0_ in patients treated with TAELE, in centimeters.

*μ*_2_ = mean tumoral size at T_0_ in patients treated with cTACE, in centimeters.

*μ*_3_ = mean difference in size of the tumor mass between T_0_ and T_2_ for TAELE group, in centimeters.

*μ*_4_ = mean difference in size of the tumor mass between T_0_ and T_2_ for cTACE group, in centimeters.

*μ*_5_ = mean tumor devascularization in the time range T_0_–T_2_ for TAELE group, in centimeters.

*μ*_6_ = mean tumor devascularization in the time range T_0_–T_2_ for cTACE group, in centimeters.

### Radiological response

Regardless of the procedure performed, a reduction in size of the tumor was found in all patients at 1-month assessment MDCT scan control, i.e. in the interval time T_0_-T_2_. Indeed, the radiological response to TAELE or cTACE was evaluated case by case according to mRECIST, as illustrated in Table 3: at 1-month assessment MDCT we registered 27/45 (60%) CR, 18/45 (40%) PR, 0 PD and 0 SD for patients treated with TAELE, and 24/42 (57.14%) CR, 18/42 (42.86%) PR, 0 PD and 0 SD for patients treated with cTACE, with no significant difference between the two procedures in terms of mRECIST radiological tumor response (p-value = 0.958), as revealed by Table 2.

**Table 3:** mRECIST Response Evaluation.

TAELE (n = 45)							
	***N***	**Males**	**Females**	**Males with AE**	**Females with AE**	**Nr of AE in males** *	**Nr of AE in females** **
**CR**	27/45 (60.00)	9/45 (20.00)	18/45 (40.00)	4/45 (8.89)	5/45 (11.11)	4/360 (1.11)	6/360 (1.67)
**PR**	18/45 (40.00)	8/45 (17.78)	10/45 (22.22)	4/45 (8.89)	6/45 (13.33)	7/360 (1.94)	11/360 (3.06)
**SD**	0/45 (0.00)	0/45 (0.00)	0/45 (0.00)	0/45 (0.00)	0/45 (0.00)	0/360 (0.00)	0/360 (0.00)
**PD**	0/45 (0.00)	0/45 (0.00)	0/45 (0.00)	0/45 (0.00)	0/45 (0.00)	0/360 (0.00)	0/360 (0.00)
**cTACE (n = 42)**							
	***N***	**Males**	**Females**	**Males with AE**	**Females with AE**	**Nr of AE in males** *	**Nr of AE in females** **
**CR**	24/42 (57.14)	10/42 (23.81)	14/42 (33.33)	3/42 (7.14)	8/42 (19.05)	4/336 (1.19)	13/336 (3.87)
**PR**	18/42 (42.86)	6/42 (14.29)	12/42 (28.57)	5/42 (11.90)	8/42 (19.05)	9/336 (2.68)	22/336 (6.55)
**SD**	0/42 (0.00)	0/42 (0.00)	0/42 (0.00)	0/42 (0.00)	0/42 (0.00)	0/336 (0.00)	0/336 (0.00)
**PD**	0/42 (0.00)	0/42 (0.00)	0/42 (0.00)	0/42 (0.00)	0/42 (0.00)	0/336 (0.00)	0/336 (0.00)

**Note.** Data in parentheses are percentages. The number of adverse events are defined considering all possible adverse events for both TAELE and cTACE groups. Adverse events (AE) belonging to CTCAE version 4.0 Grade 5 were not considered, as no casualty was registered in our series.

TAELE = transarterial ethanol-lipiodol embolization. cTACE = conventional transarterial chemoembolization. CR = Complete Response. PR = Partial Response. SD = Stable Disease. PD = Progressive Disease.

* Indicates the rate of adverse events occurred in the subgroup of males with CR, PR, SD or PD after TAELE or cTACE, respectively

** Indicates the rate of adverse events occurred in the subgroup of females with CR, PR, SD or PD after TAELE or cTACE, respectively

However, the ANOVA test displayed in Table 2 shows that TAELE resulted to be more effective than cTACE in the mean reduction of the lesion size (p-value = 0.0003). Moreover, higher degree of devascularization was obtained in patients treated with TAELE than in those treated with cTACE (p-value = 0.0004), as shown in Table 2. In particular, in patients treated with TAELE, the process of devascularization was complete in 26/45 (57.8%) and partial in 19/45 (42.2%); while in patients treated with cTACE, it was complete in 12/42 (28.6%) and partial in 30/42 (71.4%).

### Adverse Events

Protocols for trans-arterial treatments of HCC generally result in a characteristic self-limiting syndrome following embolization, which is described in 40 to 100% of patients, with abdominal pain, ileus, swinging fever, nausea, vomiting and increased serum liver enzymes (AST > 40 mg/dl; ALT > 40 mg/dl) and total bilirubine > 1.1 mg/dl, occurring hours to days after the procedure. As displayed in Table 4, CTCAE version 4.0 Grade 1 adverse effects (mostly abdominal pain and nausea) occurred in 12/45 (26.67%) patients treated with TAELE and in 20/42 (47.62%) patients treated with cTACE. Fever >38° rarely occurred in both groups and was treated with 1000mg/die paracetamol. In only one patient treated with cTACE, fever >38° reappeared twenty days after the procedure due to the development of an hepatic abscess. No patient had hepatic failure. No adverse event belonging to CTCAE version 4.0 Grade 4 was found. No death related to TAELE or cTACE did occur in our series.

**Table 4:** Toxicity.

***Adverse Event*:** GRADE 1–2		
Abdominal Pain	12/45 (26.67)	20/42 (47.62)
Fatigue/Fever	8/45 (17.78)	12/42 (28.57)
Nausea/Vomiting	3/45 (6.67)	7/42 (16.67)
***Adverse Event*:** GRADE 3–4		
Acute Renal Failure	0/45 (0.00)	0/42 (0.00)
Hypertension	0/45 (0.00)	0/42 (0.00)
Hypoxia	0/45 (0.00)	0/42 (0.00)
AST/ALT +++	5/45 (11.11)	8/42 (19.05)
Bilirubin +++	0/45 (0.00)	1/42 (2.38)
***Adverse Event*:** GRADE 5		
Mortality	0/45 (0.00)	0/42 (0.00)

**Note.** Data in parentheses are percentages. AST = aspartate aminotransferase. ALT = alanine aminotransferase.

Table 4 shows that a post-embolization syndrome was evident in our series in 24/42 (57.14%) patients treated with cTACE, while only 19/45 (42.2%) of those treated with TAELE experienced at least one symptom. As displayed in Table 2, the difference in the number of patients with embolization-related symptoms was not significant (p-value = 0.239), whereas there was a significant difference in the number of reported symptoms by each patient experiencing a post-embolization syndrome (p-value = 0.009), thus suggesting that TAELE is overall better tolerated than cTACE.

However, in order to evaluate which procedure was definitely less invasive and better tolerated, we analyzed the levels of serum transaminases and total bilirubin, as unbiased signs of damage to normal liver parenchyma.

For both TAELE and cTACE groups, Table 5 shows the number of patients with abnormal values of AST, ALT and total bilirubin, respectively at T_0_ (baseline), T_1_ (first week after the procedure) and T_2_ (one month after the procedure). Statistical tests performed to find and analyze significant variations of these parameters in the time-ranges T_0_-T_1_, T_1_-T_2_ and T_0_-T_2_ were listed in Table 2:

1. In the time-range T_0_-T_1_, no significant difference was noticed in the number of patients with abnormal values of AST, ALT or total bilirubin for TAELE group, while an increased number of patients with abnormal values of AST (p-value = 0.0112), ALT (p-value = 0.001) and total bilirubin (p-value = 0.0001) was found for cTACE group.
2. In the time-range T_1_-T_2_, a significant decrease in the number of patients with abnormal values of AST (p-value = 0.0176), ALT (p-value = 0.0059) and total bilirubin (0.0195) was detected for TAELE group, while no significant differences were found for cTACE group.
3. In the time-range T_0_-T_2_, a significant decrease in the number of patients with abnormal values of total bilirubin (p-value 0.0461) was diagnosed for TAELE group, while an increase in the number of patients with abnormal values of AST (p-value = 0.0195), ALT (p-value = 0.0107) and total bilirubin (p-value = 0.0032) was found for cTACE group.

**Table 5:** Number of patients with abnormal values of serum levels of AST, ALT and total bilirubin at T_0_, T_1_ and T_2_.

	T_0_	T_1_	T_2_	T_0_ –T_1_	T_1_-T_2_	T_0_-T_2_
**TAELE (n = 45)**						
AST	32/45 (71.11)	36/45 (80.00)	27/45 (60.00)	31/45 (68.89)	24/45 (53.33)	24/45 (53.33)
ALT	27/45 (60.00)	31/45 (68.89)	22/45 (48.89)	26/45 (57.78)	21/45 (46.67)	21/45 (46.67)
Bilirubin	27/45 (60.00)	27/45 (60.00)	20/45 (44.44)	23/45 (51.11)	19/45 (42.22)	17/45 (37.78)
**cTACE (n = 42)**						
AST	27/42 (64.29)	36/42 (85.71)	34/42 (80.95)	25/42 (59.52)	31/42 (73.81)	26/42 (61.90)
ALT	23/42 (54.76)	33/42 (78.57)	31/42 (73.81)	23/42 (54.76)	30/42 (71.43)	22/42 (52.38)
Bilirubin	14/42 (33.33)	27/42 (64.29)	24/ 42 (57.14)	14/42 (33.33)	23/42 (54.76)	13/42 (30.95)

**Note.** Data in parentheses are percentages.

T_0_ = baseline, one day before the procedure.

T_1_ = within the first week after the procedure.

T_2_ = one month after the procedure.

TAELE = transarterial ethanol embolization. cTACE = conventional transarterial chemoembolization. AST = aspartate aminotransferase. ALT = alanine aminotransferase.

### Survival outcome analysis

**Fig 1:**
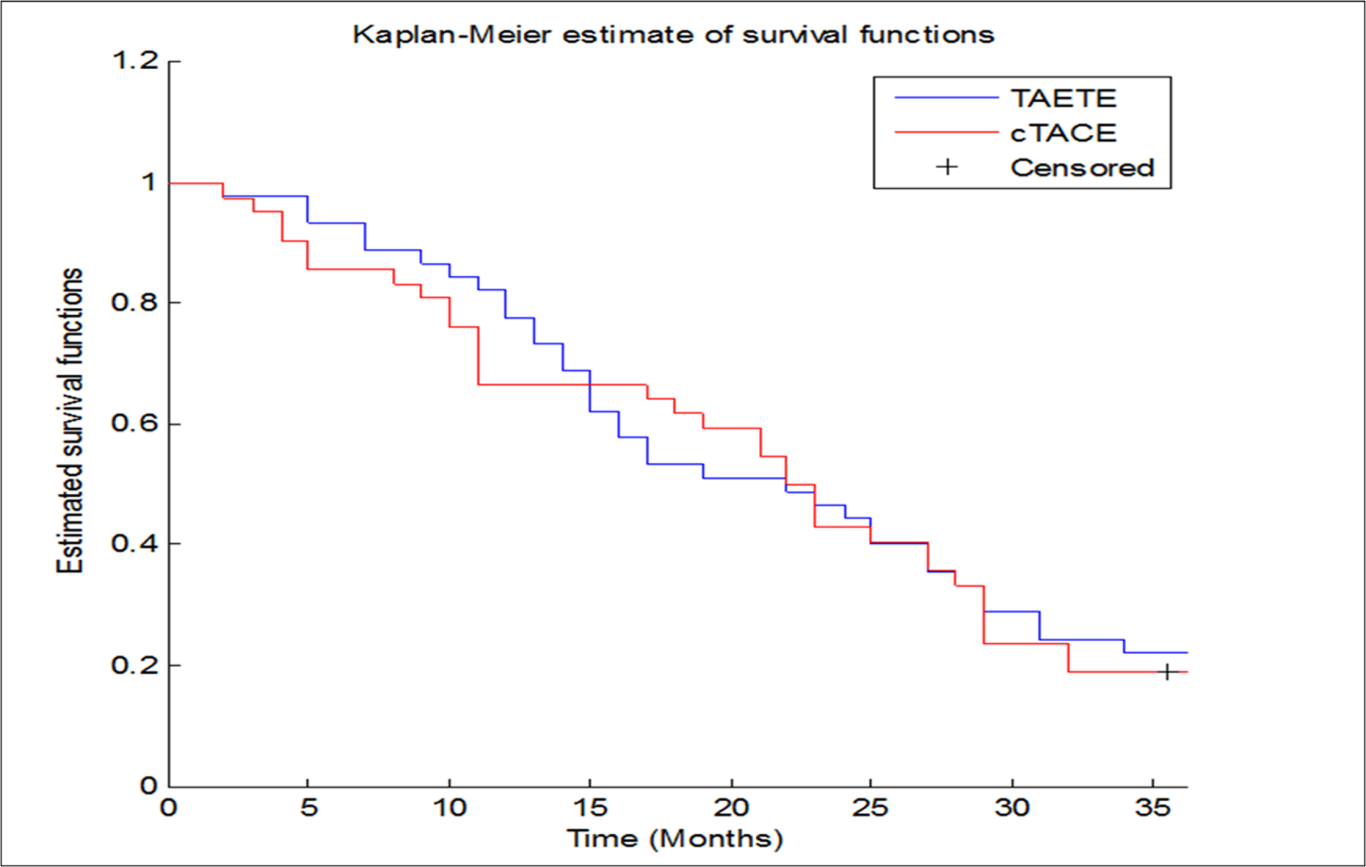
Survival curves for patients treated with TAELE and cTACE group.

Survival during 36-month follow-up was estimated for both groups and compared with Kaplan-Meier survival curves (Fig 1). No significant difference was evident using the log-rank test (p-value = 0.884). In particular, the 12th, 24th and 36th-month survival rates were 66.67%, 42.86% and 16.67%, respectively for patients treated with cTACE, and 82.22%, 46.67% and 22.22%, respectively for patients treated with TAELE.

## Discussion

HCC is a primary malignant tumor of the liver with an estimated incidence of 626,000 new patients per year [1], constantly increasing in Europe and United States [23].

Although there is no universally accepted HCC staging system, many have adopted the BCLC classification [24], also endorsed by the European Association for the Study of the Liver (EASL) and the American Association for the Study of Liver Disease [25,26]. In particular, BCLC staging system links the stage of the disease to a specific treatment strategy, such as curative treatments or palliative therapies. Very early-stage (BCLC Stage 0) and early-stage HCC (BCLC Stage A) are amenable to potentially curative therapies, such as hepatic resection, liver transplantation and percutaneous ablation (RFA) [3], providing best 5-year survival of more than 50% [27]. Transplantation is preferred by many authors because it removes underlying diseased liver that predisposes to subsequent development of new hepatic lesions [28]. Anyway, intermediate and advanced HCC (BCLC Stage B and C) are considered as unresectable, while recurrence is common in case of transplantation [29]. In such patients, palliative treatments such as TACE or systemic therapy with *Sorafenib* are recommended by BCLC as treatments of choice for prolonging survival [30].

According to BCLC guidelines, cTACE represents the standard of care for patients with intermediate stage HCC with compensated liver disease [31–33], even if a recent meta-analysis concluded that there is no firm evidence to support or refuse cTACE in patients with unresectable HCC [34]. Although cTACE prolongs survival, it is often associated with occurrence of post-embolization syndrome, thus reducing the patient tolerance of this treatment [35]. Moreover, poor quality life and severe complications are often reported [34–36].

On the other hand, the procedure that leads to the mere occlusion of the arterial flow to lesion is known as Trans-Arterial Embolization (TAE) [17]. The tumor-feeding arteries obstruction may be achieved by the injection or intra-arterial placement of embolizing agents [37]. In particular, PVA particles can cause a permanent or semipermanent arterial occlusion and achieve a more distal obstruction [38]. So far, the evidence of an additive or synergic antitumoral effect of TACE versus TAE is still unavailable. Randomized controlled trails have failed to demonstrate a significant difference in survival between the two procedures [39], suggesting that ischemia resulting from embolization might be the main factor inducing reduction in tumor size after cTACE [40].

The current study analyses a low-cost, loco-regional treatment for intermediate HCC, in which a well-tolerated mixture 1:1 of Lipiodol and Ethanol is administered directly into the tumor-feeding arteries through a selective trans-arterial approach. In the past, Ethanol has been widely used in the percutaneous approach for small unresectable HCCs [41], proving to be a safe, effective, repeatable and low-cost therapy for HCC, with lower rate of major complications, if compared to other loco-regional treatments. Embolizing procedures using Lipiodol and Ethanol have also been described for HCC: in 1993, Park et al. [9] performed this procedure on 14 male patients with single small HCC, using a mixture 3:1 of Ethanol and Lipiodol; also Cheng et al. [10] described a similar approach in 2000, on 20 patients with inoperable tumors; Cheung et al. [11] presented in 2005 a 100-patient review with 24-month follow-up and survival rates after TAE similar to those showed in the present study; more recently, a small pilot study by Gu [12] in 2010 succeeded to prove the effectiveness of trans-arterial embolization of HCC using a mixture 1:1 of Ethanol and Lipiodol, concluding that this procedure could be better than TACE in treating refractory disease. Moreover, a trans-arterial procure using Ethanol and Lipiodol has been also described in comparison with TACE in a case-controlled study by Yu et al. [13] in 2009, which assessed that the efficacy and treatment effectiveness of TAE were probably even superior to those of chemoembolization. However, to our knowledge, none of these studies focused on the procedure-related toxicity, with particular regard to post-embolization syndrome.

In our series, the mere reduction in tumor size was significantly higher in patients treated with TAELE than cTACE. This was probably due to the higher degree of devascularization achieved in lesions treated with TAELE. Indeed, it has been suggested that the embolization-related ischemia might be the main factor inducing reduction in tumor size after trans-arterial treatments [39] and that the embolizing activity could be less pronounced in cTACE than in other embolizing procedures, such as in TAELE. Anyway, the question of whether this finding means a better prognosis should be investigated in prospective studies on larger series of patients. In particular, the fact that this study is not prospective could be considered as a limitation of this study.

With regard to the adverse events, trans-arterial treatments for HCC generally result in a post-embolization syndrome [15]. In our series, embolization-related symptoms were more frequent in patients treated with cTACE than TAELE (57.14% vs 42.2%). Moreover, the elevation in serum levels of transaminases and total bilirubin was greater after cTACE than after TAELE, thus proving that TAELE is overall safe, and even safer than cTACE. Further investigations on larger populations are needed to better assess our results in terms of procedure tolerability, being the potential cumulative toxicity of embolization and chemotherapy one possible explanation.

In conclusion, in our study, TAELE showed to be more effective in tumor devascularization and size-reduction, and less toxic than conventional TACE, with similar one-month radiological outcomes according to mRECIST and similar 36-month survival. In particular, the reduced rate of adverse events makes it better tolerated in all patients, especially in those with multiple lesions, likewise in case of relapse.

All authors of this manuscript declare that they have no relationships with any company, whose products or services may be related to the subject matter of this article. All authors have nothing to disclose.
